# The Role of Melanin-Concentrating Hormone and Its Receptors in Energy Homeostasis

**DOI:** 10.3389/fendo.2013.00049

**Published:** 2013-04-22

**Authors:** Douglas J. MacNeil

**Affiliations:** ^1^Department of In Vitro Pharmacology, Merck Research LaboratoriesKenilworth, NJ, USA

**Keywords:** MCH, neuropeptide, MCHR1, orexigenic, obesity, KO mice, antagonist, clinical study

## Abstract

Extensive studies in rodents with melanin-concentrating hormone (MCH) have demonstrated that the neuropeptide hormone is a potent orexigen. Acutely, MCH causes an increase in food intake, while chronically it leads to increased weight gain, primarily as an increase in fat mass. Multiple knockout mice models have confirmed the importance of MCH in modulating energy homeostasis. Animals lacking MCH, MCH-containing neurons, or the MCH receptor all are resistant to diet-induced obesity. These genetic and pharmacologic studies have prompted a large effort to identify potent and selective MCH receptor antagonists, initially as tool compounds to probe pharmacology in models of obesity, with an ultimate goal to identify novel anti-obesity drugs. In animal models, MCH antagonists have consistently shown efficacy in reducing food intake acutely and inhibiting body-weight gain when given chronically. Five compounds have proceeded into clinical testing. Although they were reported as well-tolerated, none has advanced to long-term efficacy and safety studies.

## Introduction

**Figure 1:**
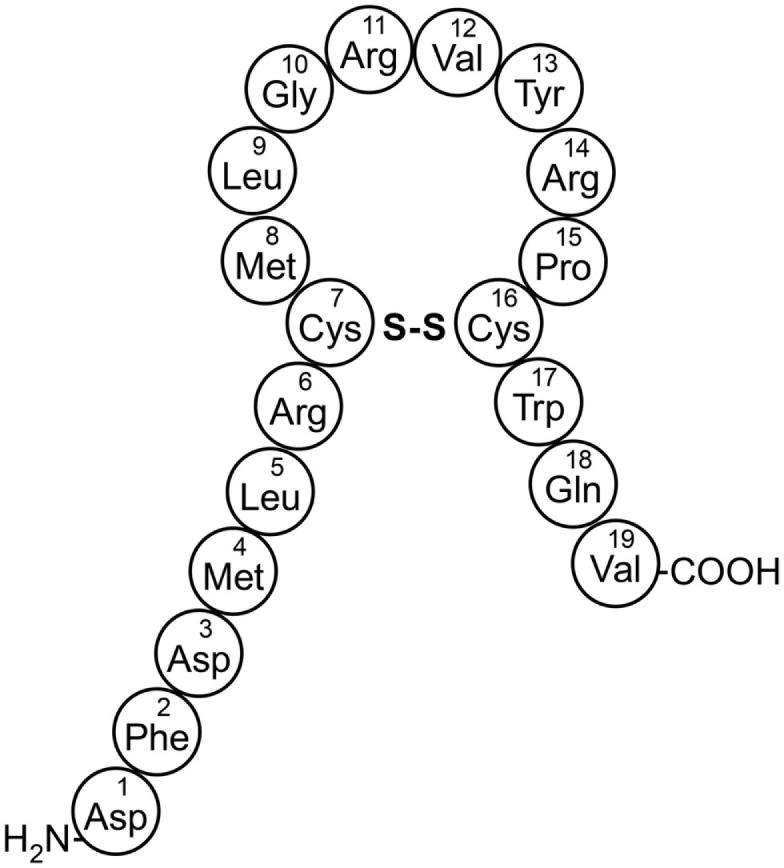
**The amino acid structure of mammalian MCH**.

The mammalian form of melanin-concentrating hormone (MCH), is a 19-amino acid cyclic peptide encoded within a 165-amino acid preprohormone (Figure 1) (Vaughan et al., 1989). MCH has been associated with a wide variety of behaviors (see recent reviews by Saito and Nagasaki, 2008; Antal-Zimanyi and Khawaja, 2009; Chung et al., 2011), but the focus of this review is the role of MCH in energy homeostasis. The amino acid sequence of MCH is identical in all mammals evaluated and alternative processing of the preproMCH peptide can generate two additional putative peptides, designated neuropeptide E-I (NEI) and neuropeptide G-E (NGE) (Nahon et al., 1989). Several *in vivo* studies have shown that MCH plays a role in a variety of physiologic processes mediated within the central nervous system (CNS), including energy homeostasis sleep and arousal, and emotionality (Yumiko and Nagasaki, 2008; Torterolo et al., 2009). Although less studied, MCH may also have a role in peripheral tissues such as in gut and pancreatic islet function (Pissios et al., 2007; Kokkotou et al., 2008).

Two MCH G-protein coupled receptors (GPCRs) have been characterized (Pissios et al., 2006; Chung et al., 2011). MCHR1 is found in all vertebrates, while MCHR2 is found in non-rodent higher species, including primates (Hill et al., 2001; Sailer et al., 2001).

Neurobiology, rodent genetics, and rodent pharmacologic studies all demonstrate that MCH and the MCH receptors are involved in regulating body weight (Gomori et al., 2003, 2007; Hervieu, 2006; Bednarek, 2007; Antal-Zimanyi and Khawaja, 2009; Johansson, 2011; Cheon, 2012). On the basis of this information, many pharmaceutical companies have pursued the development of MCHR1 antagonists for the treatment of obesity (for a recent review, see Johansson, 2011). Unfortunately, although a few MCHR1 antagonists have entered development, no compound has successfully demonstrated anti-obesity efficacy in a clinical trial. It remains unclear if this lack of clinical efficacy is due to a lack of efficacy via the MCH1R pathway or to the inability of teams to identify safe and well-tolerated compounds with sufficient potency and pharmacodynamics properties to test the hypothesis. This review summarizes the evidence for a role of MCH and its receptors in energy homeostasis and the progress made to date toward identifying small-molecule antagonists to treat obesity.

## MCH Acts through Two G-Protein Coupled Receptors

Originally described as the orphan receptor SLC-1/GPR24 (Kolakowski et al., 1996), MCHR1 was later shown by five groups to be activated by MCH (Pissios et al., 2006). The 402-amino acid rodent and human MCHR1 receptors are highly homologous, sharing ∼95% identity (Pissios and Maratos-Flier, 2003), and the highest expression of the receptors is within the brain (Saito et al., 1999; Hill et al., 2001). Like many family A GPCRs, the MCHR1 receptors have consensus *N*-glycosylation sites at the amino terminus and several potential phosphorylation sites in the intracellular loops (Lakaye et al., 1998).

In recombinant cell lines, the natural ligand, MCH, binds to MCHR1 with ∼1 nM affinity, and it couples to Gi, Go, and Gq proteins (Hawes et al., 2000; Pissios et al., 2003). Thus, activation of MCHR1 leads to an increase in intracellular Ca^++^ accumulation acting through the Gq-coupled pathway and/or to lowered cyclic adenosine monophosphate (cAMP) levels via the Gi/o-coupled pathway. Further analyses of the signaling of MCHR1 in recombinant cell lines and in brain slices demonstrates that activation of MCHR1 also leads to ERK phosphorylation (Pissios et al., 2003). In 3T3-L1 adipocytes, MCH rapidly induced a threefold to fivefold increase in MAPK pathway activities (Bradley et al., 2002). It is unclear if all, or some, of these signaling pathways contribute to MCH-mediated events *in vivo*.

A second MCHR was later identified and termed MCHR2 by six groups (Antal-Zimanyi and Khawaja, 2009). The functional role of MCHR2 is not well defined, in part because it is not expressed in rodents and related species (hamsters, guinea pigs, or rabbits), but it is expressed in humans, dogs, ferrets, and monkeys (Tan et al., 2002). The amino acid sequence identity between MCHR1 and MCHR2 is low, ∼38%, with the highest homology in the seven-transmembrane domains that form the ligand binding pocket (Sailer et al., 2001). Although MCH binds to MCHR1 and MCHR2 with a similar nanomolar affinity, the signal transduction mechanism of MCHR2 is limited to the Gq-mediated increase in intracellular Ca^++^ levels (Sailer et al., 2001). MCHR2 is largely co-expressed with MCHR1 in the CNS (Sailer et al., 2001), although peripheral expression was also found in adipocytes, pancreas, prostate, and intestine (An et al., 2001). The phylogenetic tree of MCH-related receptors contains opioid, somatostatin, galanin, urotensin 2, and orphan receptors (Sailer et al., 2001). MCH receptors have the highest homology (about 40%) with the somatostatin receptors (Sailer et al., 2001).

## Neuroanatomy of MCH and MCH Receptors

Melanin-concentrating hormone has been implicated in many behaviors. The hypothalamus is one of the primary sites in which MCH-containing nerve fibers and MCH receptors are extensively expressed (Gao, 2009). Although most of the MCH neurons are located within the incerto-hypothalamic and lateral hypothalamic area (LHA), a recent review by Bittencourt details the locations throughout the brain of MCH nerve terminals (Bittencourt, 2011). Neural signaling by MCH via its receptors has been implicated in the control of energy balance, but due to the wide distribution of MCH-containing fibers throughout the brain, the critical sites of action for particular behaviors have not been identified (Zheng et al., 2005). In male rats, neurons expressing MCH are found in the LHA and medial zona incerta, as well as, sparsely, in the olfactory tubercle and pontine reticular formation. The wide distribution of MCH fibers suggests the involvement of this neuropeptide in a variety of functions, including arousal, neuroendocrine control, and energy homeostasis (Rondini et al., 2007).

Melanin-concentrating hormone-expressing neurons in the LHA play an integrative role between signals from the periphery, acting via first-order neurons in the arcuate nucleus, and then from extra-hypothalamic systems, which modulate regulation of feeding, drinking, and seeking behaviors (Guyon et al., 2009). Factors from the periphery affect brain activity, resulting in changes in food intake and energy expenditure. Neurons from the arcuate nucleus detect changes in homeostatic parameters and transmit information to other brain areas, including the LHA. These secondary area neurons have widespread projections throughout the brain, and their activation leads to coordinated and altered behaviors (Guyon et al., 2009). About 25% of the LHA neurons projecting to the pedunculopontine tegmental (PPT) nucleus are immunoreactive for MCH, and 75% of the LHA neurons projecting to the cerebral motor cortex also contain MCH (Elias et al., 2008). Also, 15% of the incerto-hypothalamic neurons projecting to the PPT express MCH immunoreactivity. The MCH neurons express glutamic acid decarboxylase mRNA, a gamma-aminobutyric acid (GABA) synthesizing enzyme, indicating that the MCH/GABA neurons are involved in inhibitory modulation and their activation may lead to decreased motor activity in states of negative energy balance (Elias et al., 2008). Like MCH, vasopressin and oxytocin can influence energy homeostasis and other behaviors. Whole-cell recording in hypothalamic brain slices from the MCH-green fluorescent protein transgenic mouse revealed that both vasopressin and oxytocin evoked a substantial excitatory effect on MCH-expressing cells (Yao et al., 2012). Both neuropeptides reversibly increased spike frequency and depolarized the membrane potential in a concentration-dependent manner, suggesting that vasopressin or oxytocin exerts a robust excitatory effect on presumptive GABA cells that contain MCH (Yao et al., 2012).

Interestingly, projections to ventral medullary sites apparently play a role in the inhibitory effect of MCH on energy expenditure, but not food intake (Zheng et al., 2005). In the rat, a significant proportion (5–15%) of primarily perifornical and far-lateral hypothalamic MCH neurons project to the dorsal vagal complex. Retrograde tracing in the caudal brainstem demonstrated that MCH-immunoreactive axons are distributed densely in the nucleus of the solitary tract, in the dorsal motor nucleus of the vagus, and in sympathetic premotor areas in the ventral medulla (Zheng et al., 2005). In medulla slice preparations, MCH inhibited the amplitude of excitatory postsynaptic currents. Administration of MCH in the fourth ventricle in freely moving rats decreased core body temperature, but it did not change locomotor activity or food and water intake (Zheng et al., 2005).

The MCH pathways from the lateral hypothalamus to the mammillary nucleus may also enable the animal to look for food during the initial moments of appetite stimulation (Casatti et al., 2002). Injection of the retrograde tracer True Blue in the medial mammillary nucleus led to MCH/True Blue double-labeled neurons in the LHA, the rostromedial zona incerta, and the dorsal tuberomammillary nucleus. The afferents were confirmed using implants of the anterograde tracer *Phaseolus vulgaris* leucoagglutinin. The MCH projections may participate in spatial memory processing mediated by the medial mammillary nucleus (Casatti et al., 2002).

In addition to the LHA, the nucleus accumbens shell (AcSh) is a brain region important for food intake. The AcSh contains high levels of receptor for MCH. MCH receptor activation in the AcSh increases food intake, while AcSh MCH receptor blockade reduces feeding. Moreover, *in vivo* recordings confirm that MCH reduces neuronal activity in the AcSh in freely moving animals, consistent with a model from other pharmacological and electrophysiological studies whereby reduced AcSh neuronal firing leads to food intake (Sears et al., 2010). Since the AcSh mediates reinforcing properties of food, MCH may modulate motivational aspects of feeding. Indeed, chronic loss of rat MCH decreased food intake predominantly via a reduction in meal size during development and reduced high-fat food reinforced operant response in adult rats (Mul et al., 2011). Also, chronic loss of ProMCH in the rat affects the limbic dopamine system, since adult *Pmch^−/−^* rats showed increased *ex vivo* electrically evoked dopamine release (Mul et al., 2011). Thus, MCH actions in the AcSh mediate motivational aspects of feeding behavior.

MCHR1 is widely distributed in the brain (Hervieu et al., 2000; Able et al., 2009). Hervieu et al. (2000) used *in situ* hybridization histochemistry and immunohistochemistry to determine that *Mchr1* mRNA and protein were widely expressed throughout the rat brain. Similar to the distribution of MCH, *Mchr1* signals were observed in the cerebral cortex, caudate-putamen, hippocampal formation, amygdala, hypothalamus, and thalamus, as well as in various nuclei of the mesencephalon and rhombencephalon (Hervieu et al., 2000). Able et al. (2009) used an MCHR1-specific radioligand to demonstrate highly MCHR1-specific binding in the rat nucleus accumbens, caudate-putamen, and preform cortex, as well as lower levels of binding in the hippocampus and amygdala. Surprisingly, and in contrast to Hervieu et al. (2000) and Able et al. (2009) did not detect MCHR1 binding in the hypothalamus.

The distribution of MCHR2 in the primate brain nearly overlaps that of MCHR1, but the latter shows much higher relative levels and a wider distribution pattern (Mori et al., 2001). *MCHR2* is expressed in several human brain areas, including the hippocampus and amygdala, although its distribution in the hypothalamus remains controversial. Specifically, *MCHR2* mRNA was reported to be mainly expressed in the arcuate nucleus and ventromedial hypothalamic nucleus in African green monkeys by *in situ* hybridization (Sailer et al., 2001), while three other reports did not detect its expression in the human hypothalamus by RT-PCR (Hill et al., 2001; Mori et al., 2001) or Northern blot analysis (Rodriguez et al., 2001).

## Human Genetics of MCH and Energy Homeostasis

Genetic analysis of obese subjects has identified several variants of MCH and the MCH receptors, but no alterations have been conclusively linked to obesity or leanness. In an association study, among 106 subjects with severe early onset obesity and a history of hyperphagia, two missense variants were found in *MCHR1*: Y181H and R248Q (Gibson et al., 2004). Neither of these was found in 192 normal-weight controls. R248Q co-segregated with obesity across two generations, but family data were unavailable for Y181H. When tested for functional response, the R248Q variant showed no evidence of constitutive activation, alteration in cAMP signaling, or ligand hypersensitivity (Gibson et al., 2004). Two common single-nucleotide polymorphisms (SNPs) were found to be in linkage disequilibrium, but no association between either of these and obesity-related phenotypes was found among a population cohort of 541 whites. Only two rare, non-coding variants were found in *MCHR2*. However, the relationship of these *MCHR2* variants to metabolic phenotypes has not been clarified (Gibson et al., 2004). Genomic screening of 13.4 kb encompassing the *MCHR1* in extremely obese German children and adolescents identified 11 infrequent variations and two SNPs in the *MCHR1* coding sequence and 18 SNPs (eight were novel) in the flanking sequence. Although an association of an *MCHR1* haplotype (SNPs rs133072 and rs133073) with obesity was observed in two cohorts of German children and adolescents, it was not confirmed in five independent cohorts (Wermter et al., 2005). To investigate the possible polygenic role of *MCHR1*, six common SNPs (minor allele frequency >5%) found in the sequenced regions were screened in 557 morbidly obese adults, 552 obese children, and 1195 non-obese non-diabetic control subjects (Bell et al., 2005). The plausible promoter SNP, rs133068, was found to be associated with protection against obesity in obese children only (Bell et al., 2005).

A functional analysis of 11 MCHR1 variants that had been reported previously in the literature identified two mutant receptors, R210H and P377S, that failed to respond to MCH (Goldstein et al., 2010). Five other variants showed significant alterations in MCH efficacy, ranging from 44 to 142% of the wild-type value. Both inactive receptors had cell surface expression that was comparable to wild-type (Goldstein et al., 2010). It is of note that the two loss-of-function mutants were identified in markedly underweight individuals, raising the possibility that a lean phenotype may be linked to deficient MCHR1 signaling (Goldstein et al., 2010). Additional association studies with larger cohorts are needed to explore the extent to which signaling-deficient MCHR1 variants influence the maintenance of body weight.

The association between *MCHR2* variation and human obesity was investigated in 141 obese children and 24 non-obese adult subjects by DNA sequencing, and by case-control analyses using 628 severely obese children and 1401 controls (Ghoussaini et al., 2007). None of the *MCHR2* variants showed an association with adult severe obesity, but the A76A SNP was associated with severe obesity (*P* = 0.01) and overeating in obese children (*P* = 0.02) (Ghoussaini et al., 2007). Validation of an association of *MCHR2* with obesity requires replication in other cohorts.

The common allele of the *ProMCH* gene, rs7973796, may be associated with a higher body mass index (BMI) in olanzapine-treated patients with schizophrenia. In a subgroup of subjects under 50 years of age among 300 schizophrenia patients, the rs7973796 genotype was associated with an effect on BMI among patients taking olanzapine (interaction *P* = 0.025) (Chagnon et al., 2007). Olanzapine-treated patients with schizophrenia carrying the homozygote genotype showed a higher BMI for rs7973796 (*P* = 0.016 with the least-squares means *t*-test) than the variant homozygotes. The G allele was associated with an increase in the odds of obesity in schizophrenic patients taking olanzapine (Chagnon et al., 2007).

## Rodent Genetics Indicate a Role for MCH in Energy Homeostasis

Multiple mouse knockout (KO) models have been constructed to explore the role of MCH in energy homeostasis and other MCH-mediated behaviors. The KO models include multiple constructs that prevent synthesis of MCH and the MCHR1 receptor. Studies of these mice show that loss of MCH function leads to leanness and resistance to obesity.

### MCH KO mice

Strong evidence that MCH mediates energy homeostasis came from studies of mice in which the *Promch* gene was inactivated. Mice constructed with targeted inactivation of the *Promch* gene in a mixed C57BL/6 × 129SvJ genetic background had reduced body weight and leanness due to hypophagia (reduced feeding) and an increased metabolic rate, despite reduced amounts of both leptin and arcuate nucleus proopiomelanocortin mRNA (Shimada et al., 1998). Evaluation of *Promch* inactivation in pure genetic backgrounds confirmed that *Promch* deficiency increased energy expenditure and promoted increased running-wheel activity (Kokkotou et al., 2005; Zhou et al., 2005). As observed previously on a mixed background, the C57BL/6 *Promch* KO mice were hypophagic; however, the 129SvEv *Promch* KOs were hyperphagic, relative to wild-type. In both C57BL/6 and 129SvEv backgrounds, deletion of *Promch* led to reduced adiposity, attenuated weight gain, and increased locomotor activity, compared with wild-type counterparts. The relative increase in activity was greater on a high-fat diet (HFD) than on regular chow (Kokkotou et al., 2005). The lean phenotype of the *Promch* KO mice persisted as the mice aged. At 19 months, C57BL/6 *Promch^−/−^* male and female mice weighed about 25% less than their wild-type counterparts as a result of reduced fat mass in *Promch^−/−^* mice. The aged *Promch^−/−^* mice exhibited improved glucose tolerance in intraperitoneal glucose tolerance tests, were more insulin sensitive, and were more active compared with wild-type controls (Jeon et al., 2006).

Further confirmation of the role of MCH in energy homeostasis came from studies of *Promch* neuron-ablated mice, generated using toxin (ataxin-3)-mediated ablation strategy in an FVB/n background (Alon and Friedman, 2006). In these mice, the *Promch* gene is present throughout development, but 60–70% of MCH-expressing neurons degenerate in the first few weeks of life (Alon and Friedman, 2006). After 7 weeks of age, the mice developed reduced body weight, body length, fat mass, lean mass, and leptin levels. As observed in the C57BL/6 *Promch^−/−^* mice, leanness was characterized by hypophagia and increased energy expenditure. In leptin-deficient *ob/ob* mice, loss of either *Promch* or MCH-containing neurons improved obesity, diabetes, and hepatic steatosis, suggesting that MCH is an important mediator of the response to leptin deficiency (Segal-Lieberman et al., 2003; Alon and Friedman, 2006).

### *Promch* tg mice

The phenotype of *Promch* tg mice overexpressing MCH also supports a role for MCH in energy homeostasis. Ludwig et al. (2001) constructed transgenic mice that overexpressed *Promch* in the lateral hypothalamus at ∼twofold higher levels than normal mice. On an FVB background, the homozygous transgenic mice fed a HFD ate 10% more and were 12% heavier than wild-type animals. Blood glucose levels were higher both preprandially and after an intraperitoneal glucose injection, and the transgenic mice were insulin-resistant (Ludwig et al., 2001). *Promch* tg heterozygous mice on a C57Bl/6 background were hyperphagic on regular chow, heavier, and insulin-resistant, but did not have elevated blood glucose (Ludwig et al., 2001).

### *Mchr1^−/−^* mice

As the gene for preproMCH encodes two additional peptides, NEI and NGE of unknown function (Nahon et al., 1989), studies with *Mchr1* KO mice provided clarification and confirmation of the role of MCH in energy homeostasis. Three groups independently produced *Mchr1^−/−^* mice. Chen et al. (2002) reported that *Mchr1* KO mice on a C57BL/6 × 129SvJ mixed background were resistant to diet-induced obesity and had fat mass that was significantly lower in both male (4.7 ± 0.6 vs. 9.6 ± 1.2 g) and female (3.9 ± 0.2 vs. 5.8 ± 0.5 g) mice than that of the wild-type control. The mice were hyperphagic on a HFD, but had a 28% higher metabolic rate than wild-type. Both leptin and insulin levels were significantly lower in male *Mchr1^−/−^* mice than in the wild-type controls, but there were no detectable differences in glucose levels. No differences were observed between heterozygotes and wild-type mice (Chen et al., 2002). Marsh et al. (2002) observed that *Mchr1^−/−^* mice also constructed on a C57BL/6 × 129SvJ mixed background had normal body weights, yet they had reduced fat mass and were hyperphagic when maintained on regular chow. In agreement with Chen et al. (2002) they observed that *Mchr1^−/−^* mice were less susceptible to diet-induced obesity, and the KO leanness was a consequence of hyperactivity and altered metabolism (Marsh et al., 2002). A later study by Zhou et al. (2005) showed that the *Mchr1* KO mice had a dramatic 250% increase in running-wheel activity along with hyperphagia (Antal-Zimanyi and Khawaja, 2009). Astrand et al. (2004) also independently generated *Mchr1^−/−^* mice on a mixed C57BL/6 × 129SvJ background and observed that the mice had an elevated metabolic rate and were hyperactive, hyperphagic, and lean. A >12% increase in heart rate without any change in blood pressure was noted (Astrand et al., 2004). Two groups studied *Mchr1* KO mice after backcrossing onto a C57BL/6 background (Bjursell et al., 2006; Ahnaou et al., 2011). In leptin-deficient *ob/ob* mice, loss of *Mchr1* reduced adiposity (although body weights were not statistically different), improved the response in an oral glucose tolerance test (OGTT), increased spontaneous movement, and improved thermoregulation upon exposure to cold, suggesting that MCH is an important mediator of the response to leptin deficiency (Bjursell et al., 2006).

The role of MCHR2 has not been investigated in animal models. MCHR2 is absent in rodents but is present in higher species, including primates (Hill et al., 2001; Sailer et al., 2001). The role of MCHR2 might be studied in MCHR2-humanized mice, but no such model has been described. Alternatively, *in vivo* studies with MCHR1- and MCHR2-selective agonists could be used to study the role of MCHR2 in non-rodent species with a functional MCHR2. Indeed, it is surprising that *in vivo* pharmacological studies with selective ligands have not been performed, in light of the availability of MCH peptides that are potent dual MCHR1/R2 agonists, selective MCHR1 agonists, MCHR2-preferring agonists, and potent MCHR1/R2 antagonists (MacNeil and Bednarek, 2009). At this point, it is unclear if MCHR1 and MCHR2 play redundant or unique roles in MCH signaling in primates, or whether MCHR2 plays any significant role in energy homeostasis in humans.

Multiple mouse models show that disruption of the MCH system, via either the peptide ligand or the receptor, results in altered energy homeostasis (Table 1). In general, loss of MCH signaling leads to a lean, diet-induced obese (DIO)-resistant phenotype due primarily to increased energy expenditure, and in some models, lower food intake.

**Table 1:** **Mouse genetic models supporting a role for MCH in energy homeostasis**.

Genotype	Phenotype	Reference
*Promch^−/−^*C57BL/6 × 129SvJ	Hypophagic, reduced adiposity, increased metabolic rate, and reduced weight	Shimada et al. (1998)
*Promch^−/−^* C57BL/6	Normophagic, reduced adiposity, increased activity and metabolic rate, and reduced weight	Zhou et al. (2005), Kokkotou et al. (2005)
*Promch^−/−^*C57BL/6 × 129SvEv	Hyperphagic, reduced adiposity, increased activity and metabolic rate, and reduced weight	Kokkotou et al. (2005)
*Promch;ataxin-3* FVB/n	Hypophagic, reduced adiposity, increased metabolic rate, normo-activity, and reduced weight	Alon and Friedman (2006)
*Promch;ataxin-3*;*ob/ob* (FVB/nXC57BL/6)	Hyperphagic, reduced adiposity, reduced glucose, reduced steatosis, and reduced weight	Alon and Friedman (2006)
*Promch^−/−^;ob/ob* C57BL/6	Reduced adiposity, reduced glucose, increased metabolic rate, increased activity, and reduced weight	Segal-Lieberman et al. (2003)
*Promch* tg/tg FVB	Increased weight, hyperphagic on HFD, increased glucose, and insulin-resistant	Ludwig et al. (2001)
*Promch* tg/^+^ C57BL/6	Increased weight, hyperphagic on chow, normo-glucose, and insulin-resistant	Ludwig et al. (2001)
*Mchr1^−/−^* C57BL/6 × 129SvJ	Reduced adiposity, hyperphagic, hyperactive, reduced weight, higher metabolic rate, and reduced insulin	Marsh et al. (2002), Chen et al. (2002), Astrand et al. (2004), Zhou et al. (2005)
*Mchr1^−/+^* C57BL/6 × 129SvJ	No differences vs. wild-type	Chen et al. (2002)
*Mchr1^−/−^* C57BL/6	Reduced insulin, improved OGTT, and elevated body temperature	Bjursell et al. (2006), Ahnaou et al. (2011)
*Mchr1^−/−^*;*ob/ob* C57BL/6	Decreased adiposity, reduced insulin, improved OGTT, increased activity	Bjursell et al. (2006)

## Pharmacologic Studies Confirm a Role for MCH in Energy Homeostasis

*In vivo* studies have shown that MCH or MCH analogs increase food intake and body weight, while, conversely, studies with MCHR1 antagonists reduce body weight and associated comorbidities.

### *In vivo* effects of MCH peptide agonists

Melanin-concentrating hormone, and occasionally MCH derivatives, have been used to evaluate the role of MCH in energy homeostasis. Most studies have utilized acute injections of MCH into either specific brain nuclei or, more commonly, the third or fourth ventricle. These injections almost certainly lead to supraphysiologic levels of MCH in at least some of the MCH receptor-containing nuclei. Qu et al. (1996) were the first to show that intracerebroventricular (ICV) MCH increased food intake. In multiple experiments ICV injections of 5 or 30 μg into Long Evans rats increased 2-, 4-, and 6-h food intake between 150 and 200% (Qu et al., 1996). ICV injection of MCH into the third ventricle of either Wistar or Sprague-Dawley rats also increased food intake (Qu et al., 1996; Della-Zuana et al., 2002; Shearman et al., 2003). The orexigenic effects of ICV MCH were maximal at 2 h post injection (Della-Zuana et al., 2002). In Wistar rats, low doses (0.1 and 0.5 μg/rat) were ineffective, while higher doses (1, 5, and 10 μg/rat) were equally effective, leading to an approximate doubling of food intake over 2–4 h (Della-Zuana et al., 2002). In Sprague-Dawley rats, only the two highest doses led to significant increases in food intake (Della-Zuana et al., 2002). When presented with sucrose solutions after ICV injection of MCH (2 nmol), Sprague-Dawley rats increased their intake of sucrose solution by increasing the rate of licking (Baird et al., 2006). The role of MCH in energy homeostasis was also confirmed in sheep, which have both MCHR1 and MCHR2; acute ICV doses of MCH increased food intake (Whitlock et al., 2005). Potent MCH analogs (IC_50_ < 25 nM), but not the weak analogs (IC_50_ > 1000 nM), reduced 2-h food intake after ICV administration of 4.4 nmol to Wistar rats (Suply et al., 2001). In a separate study in Sprague-Dawley rats using a smaller, potent MCH analog, Shearman et al. (2003) observed dose-dependent increases in 6-h food intake after an ICV injection of an MCH agonist (1 μg/rat, +68%; 5 μg/rat, +76%; 15 μg/rat, +122%). Guesdon et al. (2008) confirmed that the smaller MCH analog, when given ICV at 5 μg/rat, increased food intake in Wistar rats threefold over a 2-h period.

Intracerebroventricular injection of MCH into the third ventricle of rats significantly increased the ingestion of sucrose and glucose solution, but not of saccharin, indicating that the MCH-induced dipsogenic response is more related to caloric content than to sweet taste *per se* (Sakamaki et al., 2005). Injections of MCH into several brain nuclei led to increases in food intake. MCH (0.6 nmol) elicited a rapid and significant increase in feeding in satiated rats following injection into the arcuate nucleus, the paraventricular nucleus, or the dorsomedial nucleus (Abbott et al., 2003). However, no significant alteration in feeding was observed following injection into other brain regions associated with energy homeostasis, including the supraoptic nucleus, LHA, medial preoptic area, anterior hypothalamic area, or ventromedial nucleus of the hypothalamus (Abbott et al., 2003).

In a side-by-side comparison, MCH was found to be a weaker orexigen than two other hypothalamic neuropeptides. In lean rats, 1 and 3 nmol of the ICV-injected orexigenic peptides, neuropeptide Y (NPY) and agouti-related protein (AGRP), showed robust increases in intake of a sucrose solution, but 3 nmol of MCH mediated only a non-significant trend toward increased feeding (Semjonous et al., 2009). The importance of forebrain hypothalamic regions for MCH action was apparent when injection of 6 nmol of MCH into the fourth ventricle of lean rats or sheep failed to induce increased food intake, while control injections of NPY did increase food intake (Whitlock et al., 2005; Baird et al., 2007). Administration of LiCl, a potent inducer of conditioned taste aversion (CTA), to rats leads to an upregulation of *Mch* and *Mchr1* mRNA (Mitra et al., 2012). However, when MCH was injected prior to the induction of CTA with LiCl, as well as later during the CTA retrieval, MCH treatment did not reduce the magnitude of CTA upon subsequent presentations of the aversive tastant (Mitra et al., 2012). Thus, MCH is not critical to the development of CTA.

Although most ICV studies leading to an increase in food intake utilized injection into the third ventricle, Georgescu et al. (2005) demonstrated that direct injection of 1 μg of MCH into the AcSh, a region rich in MCHR1, resulted in a robust increase in food intake by Sprague-Dawley rats lasting at least 4 h. Guesdon et al. (2008) used a potent, truncated MCH analog and also observed that injection of 5 μg into the AcSh of Wistar rats increased food intake of regular chow threefold during the 2 h following injection.

Agonist studies in preproMCH-deficient rats confirmed that the orexigenic actions of MCH are independent of two other preproMCH encoded peptides, NGE and NEI. Acute AcSh administration of NGE and NEI, or chronic ICV infusion of NEI, did not affect feeding behavior in adult *Promch*^+*/*+^ or *Promch^−/−^* rats (Mul et al., 2011). However, acute administration of MCH to the AcSh of adult *Promch^−/−^* rats elevated feeding behavior toward wild-type levels (Mul et al., 2011).

In addition to the effects of MCH on food intake, the role of MCH in mediating energy expenditure was also compared with that of two other orexigenic peptides, AGRP and orexin. Both AGRP and orexin, administered ICV (1 nmol/mouse), significantly decreased oxygen consumption compared with artificial cerebrospinal fluid (aCSF) treated controls; in contrast, MCH (1 nmol/mouse) had no significant effect compared with aCSF-treated controls (Asakawa et al., 2002). However, an effect of MCH on oxygen consumption might not have been detected, since only a relatively low dose of peptide was tested.

Chronic ICV infusions of MCH into rodents were shown to not only increase food intake, but to also cause obesity (Della-Zuana et al., 2002; Gomori et al., 2003; Ito et al., 2003); while MCH-induced insulin resistance in rats was observed acutely in the absence of weight changes (Pereira-da-Silva et al., 2005). Chronic infusions of MCH (8 μg/rat/day) over 12 days led to an increase in body weight of about 20 g more than did control aCSF infusions in both Wistar or Sprague-Dawley rats (Della-Zuana et al., 2002). After a 14-day infusion of MCH into the third ventricle of C57BL/6J mice (10 μg/day), no significant increase in food intake was observed in mice fed a regular chow, but on a moderately high-fat diet (MHF), the mice ate about 15% more food (Gomori et al., 2003). Mice on both diets were significantly heavier than control mice, with the largest increase in body weight observed in the MCH infused mice on a MHF diet; these mice gained 17% more weight than the control infused mice on an MHF diet (Gomori et al., 2003). Glick et al. (2009) also observed that chronic infusion of 10 μg/day of MCH for 14 days into C57BL/6 mice fed regular chow led to a 34% increase in food intake and a 15% increase in body weight after a 14-day infusion. In a separate study, C57BL/6J mice on an MHF diet infused with a lower amount of MCH (3 μg/day for 7 days) also were hyperphagic and gained 350% more weight than did the vehicle control mice, with no detectable changes in activity (Ito et al., 2003). In addition, a small, potent, MCH analog given chronically ICV (30 μg/day) to Sprague-Dawley rats increased food intake by 23% and body weight by 38% more than in the vehicle controls (Shearman et al., 2003).

Intracerebroventricular-injected MCH has metabolic effects beyond increases in food intake and body weight. The acute effects of single MCH injections probably identify direct effects from increased MCH signaling in the brain, while the chronic effects may be subsequent to increased adiposity associated with body-weight gain. A single ICV injection of MCH into Wistar rats (3 nmol) inhibited the thyroid axis (Kennedy et al., 2001) by suppressing release of thyroid hormone from the hypothalamus, leading to a suppression of plasma thyroid-stimulating hormone (Kennedy et al., 2001). ICV injections of 4 nmol of MCH into Wistar rats for 4 days resulted in an ∼10% increase in fasting plasma glucose (Pereira-da-Silva et al., 2005). Guesdon et al. (2008) used a potent, truncated MCH analog and found that injection of 5 μg into the AcSh or the third ventricle of Wistar rats did not significantly increase energy expenditure, but it did increase glucose oxidation and reduce lipid oxidation in the period 3 h after injection.

Chronic ICV infusions of MCH into C57BL/6J mice resulted in increased fat pad weights (∼100%), leptin (∼300%), liver triglycerides (∼120%), fasting plasma glucose (∼10%), and insulin (∼100%) (Gomori et al., 2003). Glick et al. (2009) also observed that chronic infusion of 10 μg/day of MCH for 14 days into C57BL/6 mice led to a 4.5-fold increase in adiposity, about a 0.4°C drop in body temperature during the active dark phase, a 15% decrease in oxygen utilization, and a 26% increase in plasma insulin-like growth factor 1 levels. Chronic MCH agonist infusions into Sprague-Dawley rats also led to increases in insulin (∼200%) and leptin (∼400%) (Shearman et al., 2003).

### *In vivo* effects of MCH receptor antagonists

As discussed above, rodent genetic models clearly show a role for MCH in energy homeostasis. However, the applicability of these studies to understanding the physiological role and disease states associated with MCH signaling may be limited due to developmental effects of MCH inactivation or neuronal loss affecting other neuromodulators. Moreover, the relevance of *in vivo* pharmacology studies is complicated, because they utilize MCH and MCH agonists administered ICV at what are probably supraphysiologic levels. To better understand the role of MCH in energy homeostasis, a series of *in vivo* studies utilized MCHR1-specific antagonists. Because many pathways can affect food intake and body weight, *in vivo* effects of MCHR1 antagonists might be due to non-specific effects; however, in a few cases, researchers have demonstrated MCHR1 specificity by showing that an antagonist is inactive in *Mchr1^−/−^* mice.

#### Studies with MCHR1 peptidic antagonists

Studies on truncated and substituted MCH analogs identified key amino acids for activation of the MCH receptors and led to synthesis of potent antagonist derivatives (Bednarek et al., 2002; Audinot et al., 2009). Several *in vivo* studies employed Ac-(Ava^9–10^, Ava^14–15^)-MCH(6–16)-NH_2_, a truncated, cyclic MCH analog containing γ-aminovaleric acid which is a potent (Kb 4 nM) antagonist (MacNeil and Bednarek, 2009). Shearman et al. (2003) reported that the peptide antagonist given ICV (10 μg) did not significantly reduce spontaneous feeding, affect feeding duration or locomotor activity, or alter overnight body-weight gain in lean male Sprague-Dawley rats. However, the peptide antagonist blocked the initial 2- or 3-h hyperphagic activity of an MCH agonist (Shearman et al., 2003; Mashiko et al., 2005). Chronic ICV infusion of the antagonist at 48 μg/day for 14 days reduced cumulative food intake by 13% and body-weight gain by 33%, relative to vehicle controls (Shearman et al., 2003). In male C57BL/6J mice fed an MHF diet, 28-day ICV infusion of the peptide antagonist at 7.5 μg/day decreased cumulative food intake 15% and the antagonist-treated mice weighed 13% less than the vehicle-infused controls (Mashiko et al., 2005). The antagonist treatment led to improvements in other metabolic parameters, including reductions in plasma glucose, leptin, insulin, and total cholesterol, as well as reductions in fat pad and liver weights (Mashiko et al., 2005). The specificity of the MCHR1 peptide antagonist was confirmed using *Mchr1^−/−^* mice, as infusion of the antagonist for 2 weeks had no effect on body weight, fat mass, or cumulative food intake in the KO mice (Mashiko et al., 2005). In contrast to *Mchr1* KO mouse models (Table 1), no changes were noted in activity after a 4-week infusion of the MCHR1 antagonist (Mashiko et al., 2005). Obese, aged, male C57BL/6J mice fed a HFD for 1 y (body weight ∼60 g) were subjected to a 4-week ICV infusion with the peptide antagonist at 7.5 μg/day (Ito et al., 2008). The antagonist-infused mice lost 21% of their weight, while vehicle-infused mice gained 6% more weight. Not surprisingly, given the large differences in body weight, the MCHR1 antagonist-treated mice showed significant reductions in serum leptin and insulin (Ito et al., 2008). Also, the liver weights of the antagonist-treated DIO mice decreased by about 50%, while the serum liver markers showed improvements (Ito et al., 2008). In a follow-up study to explore the role of MCH in modulating accumulation of triglycerides in liver, male C57BL/6J mice were fed a diet deficient in methionine and choline to induce steatohepatitis; ICV treatment with the MCHR1 antagonist (7.5 μg/day for 10 days) did not result in any body-weight difference vs. vehicle control, but accumulation of triglycerides in liver was reduced 33% (Ito et al., 2008). The ability of MCHR1 antagonist treatment to improve hepatic steatosis was confirmed in a female model of obesity in which ovariectomized mice were fed a regular chow diet. Thirty weeks after the ovariectomy, the female C57BL/6J mice weighed about 25% more than did the sham treated mice (Gomori et al., 2007). When these obese mice were treated ICV with the MCHR1 peptide antagonist for 4 weeks at 7.5 μg/day, they lost 13% of their body weight and 32% of their liver triglycerides (Gomori et al., 2007).

Studies with S38151 in multiple rodent models of obesity resulted in significant effects on food intake and body weight. S38151 [*p*-guanidinobenzoyl-[des-Gly^10^]-MCH(7–17)] is a modified and truncated 11-amino acid MCH analog which is a potent (Kb = 4 nM) MCHR1 antagonist (Audinot et al., 2009). Injection of this peptidic MCHR1 antagonist into the AcSh reduced food intake (Georgescu et al., 2005). Over a 6-h period, in male Wistar rats, S38151 administered ICV dose dependently inhibited the orexigenic effects of previously injected MCH (Audinot et al., 2009). The highest doses of S38151, 30 and 50 nmol per rat, completely blocked MCH-induced food intake for 6 h (Audinot et al., 2009). The orexigenic effect of proMCH (131–165), which is more potent than MCH in stimulating feeding, was blocked for 2 h by 50 nmol/kg of S38151 administered ICV (Maulon-Feraille et al., 2002; Della-Zuana et al., 2012). Once daily ICV injections of 20 nmol/kg of S38151 into male Zucker *fa/fa* rats, reduced food intake, water intake, motility, and body weight (Della-Zuana et al., 2012). A single injection of 20 μmol/kg of S38151 intraperitoneally (i.p.), reduced cumulative food intake in Zucker *fa/fa* rats for 24 h (Della-Zuana et al., 2012). S38151 was administered i.p. at 30 mg/kg for 5 days into two mouse models of obesity, female *ob/ob* and female C57BL/6J DIO mice, resulting in reductions in body weight and cumulative food intake. The S38151 effects on energy homeostasis were MCH1R based, since no changes in food intake or body weight were observed after 5 days of i.p. injection into female *Mchr1* KO mice (Della-Zuana et al., 2012).

#### Studies with non-peptide MCHR1 antagonists

The overwhelming set of genetic and physiologic data demonstrating that MCHR1 modulates energy homeostasis attracted the interest of a many medicinal chemistry groups who have synthesized numerous structurally distinct MCHR1 antagonists (see a recent review by Cheon, 2012). In all, 23 different companies have published more than 100 medicinal chemistry papers and patents describing attempts to optimize MCHR1 antagonist hit compounds toward drug candidates (see the recent review of the patent literature by Johansson, 2011). Many of the resulting optimized lead compounds, representing a diverse set of non-peptide MCHR1 antagonists, have been evaluated *in vivo*. Non-peptide MCHR1 antagonists are effective in different models of acute food intake in a variety of different rodent strains (Table 2) (Borowsky et al., 2002; Takekawa et al., 2002; Huang et al., 2005; Palani et al., 2005; McBriar et al., 2006; Sasikumar et al., 2006; Xu et al., 2006; Balavoine et al., 2007; Kowalski and Sasikumar, 2007; Moriya et al., 2009; Nagasaki et al., 2009; Haga et al., 2011; Kamata et al., 2011; Kasai et al., 2011, 2012). In general, MCHR1 antagonists potently block up to 75% of MCH-induced food intake (Borowsky et al., 2002; Takekawa et al., 2002; Moriya et al., 2009; Nagasaki et al., 2009), but they have more modest effects on reducing fasting-induced feeding and spontaneous feeding.

**Table 2:** **Acute effects of non-peptide MCH1R antagonists on food intake**.

Feeding model*	Time of measurement			Reference
	2 h	4, 5, or 6 h	24 h	
**MCH-INDUCED HYPERPHAGIA IN RAT**				
Male Sprague-Dawley		−90%		Takekawa et al. (2002)
Male Sprague-Dawley	−72%			Moriya et al. (2009)
Male Sprague-Dawley	−75%	−75%		Nagasaki et al. (2009)
Male Wistar	−70%			Borowsky et al. (2002)
**FASTING-INDUCED FEEDING**				
Male Wistar rats	−35%	−30%	−25%	Huang et al. (2005)
Mice (strain and gender unknown)	−80%	−40%		Balavoine et al. (2007)
DIO male C57BL/6NCrl:BR mice	−35%	−32%	−22%	McBriar et al. (2006)
DIO mice (strain and gender unknown)		−17%	−14%	Palani et al. (2005)
DIO mice (strain and gender unknown)	−12%	−20%	−9%	Sasikumar et al. (2006)
**SPONTANEOUS FEEDING**				
Male Sprague-Dawley rats			−30%	Kamata et al. (2011)
Female KK*Ay* mice	−63%			Kamata et al. (2011)
DIO C57BL/6J mice (gender unknown)			−19%	Haga et al. (2011)
DIO mice (strain and gender unknown)			−21%	Xu et al. (2006)
DIO male F344/Jcl rats			−30%	Kasai et al. (2012)
DIO male F344/Jcl rats		−28%		Kasai et al. (2011)
DIO male Sprague-Dawley rats			−31%	Kowalski and Sasikumar (2007)
Male Sprague-Dawley rats, ingestion of condensed milk^#^	−45%			Borowsky et al. (2002)

*Data shown for the most potent analog in the cited reference at the highest dose tested.

*^#^Measured at 20 min*.

Multiple MCHR1 antagonists have also shown dose-dependent and sustained efficacy in chronic models of obesity (Kym et al., 2005; Souers et al., 2005a,b, 2007; Vasudevan et al., 2005a,b; Carpenter et al., 2006; Hertzog et al., 2006; Tavares et al., 2006a,b; Mendez-Andino and Wos, 2007; Mendez-Andino et al., 2007; Gehlert et al., 2009; Ito et al., 2009; Semple et al., 2009; Suzuki et al., 2009; Hadden et al., 2010; Mihalic et al., 2012; Sasmal et al., 2012a,b). At the highest dose tested in DIO mice, ranging from 10 to 100 mpk, weight loss ranged from 5% at 5 days to 33% at 238 days (Ito et al., 2009; Mihalic et al., 2012). In several cases, weight loss was shown to be primarily due to the loss of fat mass (Souers et al., 2005a,b, 2007; Vasudevan et al., 2005a; Mendez-Andino et al., 2007). The mechanism of weight loss appears to involve a combination of reduced food intake, which was observed in six studies (Table 3), and increased energy expenditure with no increase in activity (Kowalski et al., 2006; Gehlert et al., 2009).

**Table 3:** **Chronic effects of non-peptide MCH1R antagonists in rodents**.

Rodent model of energy homeostasis*			

	Reduced food intake vs. vehicle (%)	Time (days)	Reference
**REDUCED CUMULATIVE FOOD INTAKE IN MICE**			
Male DIO C57BL/6J	16	5	Ito et al. (2009)
DIO (strain and gender unknown)	11	10	Mendez-Andino et al. (2007)
DIO (strain and gender unknown)	37	13	Kym et al. (2005)
DIO (strain and gender unknown)	13	14	Souers et al. (2005b)
Male DIO C57BL/6NCrl:BR	18	28	Kowalski et al. (2006)
**REDUCED CUMULATIVE FOOD INTAKE IN RATS**			
DIO Long Evans (gender unknown)	16	14	Gehlert et al. (2009)

	**Weight loss vs. vehicle (%)**	**Time (days)**	**Reference**

**REDUCED BODY WEIGHT IN MICE**			
DIO C57BL/6J male	5	5	Ito et al. (2009)
DIO C57BL/6J male	6	5	Surman et al. (2010)
DIO C57BL/6J male	6	6	Hadden et al. (2010)
DIO (strain and gender unknown)	8	7	Mendez-Andino and Wos (2007)
DIO (strain and gender unknown)	5	10	Mendez-Andino et al. (2007)
DIO AKR/J (gender unknown)	13	12	Hertzog et al. (2006)
DIO (strain and gender unknown)	18	13	Kym et al. (2005)
DIO C57BL/6J male	8	13	Suzuki et al. (2009)
DIO C57BL/6J (gender unknown)	6	14	Vasudevan et al. (2005b)
DIO C57BL/6J (gender unknown)	12	14	Sasmal et al. (2012a)
DIO C57BL/6J (gender unknown)	13	14	Sasmal et al. (2012b)
DIO (strain and gender unknown)	7	14	Souers et al. (2007)
DIO (strain and gender unknown)	23	14	Vasudevan et al. (2005a)
DIO (strain and gender unknown)	15	14	Souers et al. (2005a)
DIO (strain and gender unknown)	17	14	Souers et al. (2005b)
DIO AKR/J (gender unknown)	10	15	Carpenter et al. (2006)
DIO AKR/J (gender unknown)	10	21	Tavares et al. (2006b)
DIO AKR/J (gender unknown)	15	26	Tavares et al. (2006a)
DIO (strain and gender unknown)	31	28	Kym et al. (2005)
DIO C57BL/6NCrl:BR male	16	28	Kowalski et al. (2006)
DIO C57BL/6J (gender unknown)	9	137	Mihalic et al. (2012)
DIO female (strain unknown)	33	238	Mihalic et al. (2012)
**REDUCED BODY WEIGHT IN RATS**			
DIO Long Evans (gender unknown)	15	14	Gehlert et al. (2009)
DIO Sprague-Dawley male	15	14	Dyck et al. (2006)
DIO Wistar female	17	28	Semple et al. (2009)

*Data shown for the most potent analog in the cited reference at the highest dose tested.

As shown above with peptides, and in Tables 2 and 3 with non-peptide compounds, many different MCHR1 antagonists have demonstrated modest to robust efficacy in a variety of rodent obesity models, suggesting that MCHR1 antagonists may have potential for treating human obesity. One caveat for the data is that in most cases, the antagonists have not demonstrated the ability to induce weight loss through an MCHR1-specific mode of action, since efficacy in *Mchr1* KO mice has not been evaluated. In fact, in one report of robust weight loss, the authors caution that some weight loss at the highest dose tested maybe due to non-specific mechanisms, since the compound resulted in high brain levels, 6.46 μg/g (Kym et al., 2005). However, in four reports, the MCHR1-specific efficacy of antagonists was confirmed, since the antagonists lacked efficacy in DIO *Mchr1* KO mice, but they showed body-weight loss in DIO wild-type mice (Gehlert et al., 2009; Della-Zuana et al., 2012; Mashiko et al., 2005; Mihalic et al., 2012). Further support for an MCHR1 mechanism based-weight loss has been reported for three compounds, for which efficacy was correlated with brain MCHR1 receptor occupancy (Hervieu et al., 2003; Kowalski et al., 2006; Ito et al., 2009).

#### Studies with MCHR2 antagonists

The absence of the *Mchr2* in rodents has limited the interest in developing MCHR2-selective compounds. Only one paper has disclosed a potent MCHR2-selective non-peptide small-molecule antagonist (Chen et al., 2012). Compound 38 is a potent (Ki 13 nM) and selective MCHR2 antagonist with good oral bioavailability and pharmacokinetics suitable for *in vivo* studies (Chen et al., 2012). However, no *in vivo* data have been reported with this compound.

### Clinical studies with MCHR1 antagonists

**Figure 2:**
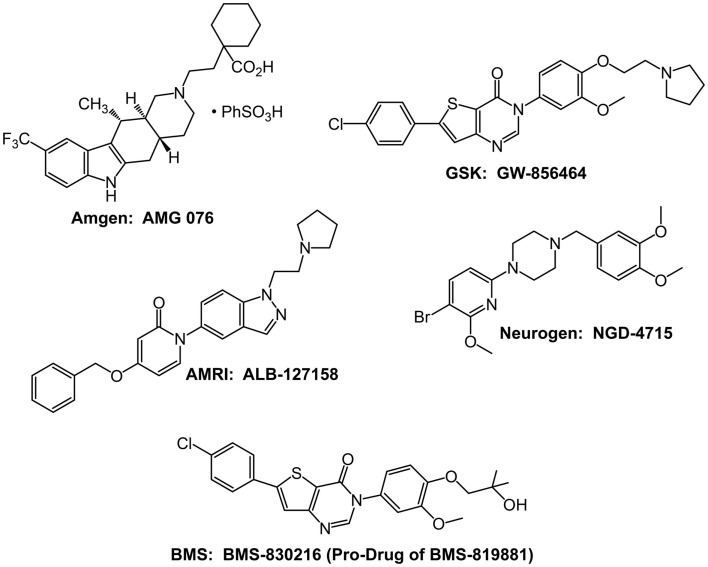
**Structures of the five MCHR1 antagonists that have been evaluated in Phase I safety and tolerability studies**.

The intense interest in MCHR1 antagonists has resulted in more than 80 publications and 100 patent applications describing various unique antagonists (Johansson, 2011). Five compounds have reached testing in human subjects, but none has proceeded into advanced Phase II studies to rigorously test their efficacy in causing chronic weight loss (Figure 2). A major issue with many lead compounds is increased cardiovascular risk due to high-affinity hERG binding and drug-induced QTc prolongation (Mendez-Andino and Wos, 2007).

The Amgen MCHR1 antagonist AMG 076 entered Phase I safety and tolerability testing in 2004, but there have been no subsequent reports of its status since 2005. GlaxoSmithKline MCHR1 antagonist GW-856464 also entered Phase I studies in 2004 (Cheon, 2012). However, on August 24, 2010, Carmen Drahl reported via Twitter that “low bioavailability precluded further development.”

NGD-4715 is a selective MCHR1 antagonist developed by Neurogen. In a May 2, 2007 press release, Neurogen announced that NGD-4715 was safe and well-tolerated in a Phase I clinical trial. Neurogen was acquired by Ligand Pharmaceuticals in 2009; as of February 22, 2013, the Wikipedia entry for NGD-4715 quotes an e-mail from Ligand that there are “no plans for further development on the compound.” In a May 31, 2011 press release, AMRI announced that its MCHR1 antagonist, ALB-127158(a), was well-tolerated in a Phase I single ascending-dose study and 14-day multiple ascending-dose safety and tolerability study. An encouraging result reported by some subjects was loss of appetite. Despite the reported tolerability and suggestion of efficacy, in a subsequent press release dated October 4, 2011, AMRI announced that development was terminated before the initiation of Phase II studies.

Bristol–Myers Squibb (BMS) conducted the longest clinical trial with an MCHR1 antagonist. As of February 22, 2013, the BMS website indicates BMS-830216 was evaluated in a 28-day Phase I study to assess the safety, tolerability, and effect on body weight and other obesity-related factors of different doses of BMS-830216. A summary of the clinical results with BMS-830216 is available on the NIH website (http://www.ncats.nih.gov/files/BMS-830216.pdf), which indicates that BMS-830216 is a prodrug of the antagonist BMS-819881 and a potent (*K*_i_ = 10 nM) and selective MCHR1 antagonist. BMS-830216 was generally safe and well-tolerated at all doses in the Phase I study for up to 28 days. However, no indications of weight loss or reduced food intake were observed, and the compound did not proceed to Phase II studies.

## Future Prospects

A rich literature of rodent genetics and rodent pharmacology demonstrates a significant role for MCH, acting via the MCHR1 within the hypothalamus, in maintaining energy homeostasis. This knowledge has stimulated more than 20 companies to seek MCHR1 selective compounds for the treatment of obesity. Five companies are known to have succeeded in identifying development candidates that proceeded through preclinical safety studies and enabled Phase I clinical safety and tolerability testing. Three of the compounds, NGD-4715, ALB-127158(a), and BMS-830216 (Figure 1), were found generally safe and well-tolerated in the Phase I studies. However, BMS-830216, which was tested for 28 days in obese subjects, failed to show any significant weight loss efficacy. No compounds have proceeded into Phase II studies in which chronic efficacy could be evaluated.

After almost 15 years of research, studies have failed to detect anti-obesity efficacy with MCHR1 antagonists in the clinic. There are multiple reasons that might account for this disconnect between rodent and human studies. First, compounds tested in the clinic may not have had the appropriate properties to sufficiently block the MCHR1 receptor and achieve an effect of energy balance. For instance, the MCHR1 antagonists may not have reached the hypothalamic sites of MCH action. Since there have been no reported studies measuring the level of receptor occupancy of the clinical compounds, it is possible that the compounds failed to sufficiently penetrate the brain: blood barrier resulting in low and insufficient receptor occupancy. Second, studies utilizing a positron emission tomography (PET) ligand demonstrated that an NPY5R antagonist must achieve >90% receptor occupancy sustained over 24 h to cause weight loss (Erondu et al., 2006). Similarly, in DIO mouse studies, maximum weight loss was observed only when an MCHR1 antagonist blocked >90% of the MCHR1 receptors for 24 h (unpublished data by the author). Clearly, a MCHR1 PET ligand could guide compound dose selection to assure 24 h of high receptor blockade (Erondu et al., 2006; Philippe et al., 2012). Third, since humans also express MCHR2 in the hypothalamus (Sailer et al., 2001), MCH signaling in humans may involve both MCHR1 and MCHR2, such that blockade of the MCHR1 alone may not be sufficient to achieve efficacy in obesity. Fourth, the role of MCH in human energy homeostasis may not be as significant as its role in rodents, consequently, blockade of MCHR1 would be inherently ineffective as a treatment for obesity.

In the author’s opinion, there is, at best, only a modest probability that an MCHR1 antagonist will be developed as a treatment for obesity. Following the failure of five Phase I compounds to progress to Phase II efficacy studies, the enthusiasm for MCHR1 antagonists has clearly dimmed. Moreover, after more than a decade of drug discovery effort by more than 20 companies, there are currently no known MCHR1 antagonists in the clinic for obesity. Should any company continue to seek MCHR1 antagonists, the author suggests that they should proceed only with the aid of a PET ligand, perhaps [^11^C]SNAP-7941, or another on-target biomarker to assess receptor occupancy (Philippe et al., 2012). Companies might also consider focusing their chemistry efforts on identifying a dual MCHR1 and MCHR2 antagonist, to assure that all MCH-mediated signaling involved in energy homeostasis is effectively blocked. Finally, an effective MCH antagonist therapy for obesity must not only achieve meaningful weight loss, but it must also be well-tolerated. Although not discussed in this review, there is significant rodent genetic and pharmacologic data indicating that MCH participates in other CNS behaviors (Saito and Nagasaki, 2008; Yumiko and Nagasaki, 2008; Antal-Zimanyi and Khawaja, 2009; Torterolo et al., 2009; Chung et al., 2011). In particular, studies in multiple rodent models suggest blockade of MCH signaling may be anxiolytic (Antal-Zimanyi and Khawaja, 2009; Chung et al., 2011). Tolerability may be a substantial hurdle for an effective MCH receptor antagonist to overcome.

## Conflict of Interest Statement

The author is employed by a company with a goal of developing novel therapies.
